# Inflammatory Drivers of Cardiovascular Disease: Molecular Characterization of Senescent Coronary Vascular Smooth Muscle Cells

**DOI:** 10.3389/fphys.2020.00520

**Published:** 2020-05-25

**Authors:** Stevan D. Stojanović, Maximilian Fuchs, Meik Kunz, Ke Xiao, Annette Just, Andreas Pich, Johann Bauersachs, Jan Fiedler, Daniel Sedding, Thomas Thum

**Affiliations:** ^1^Institute of Molecular and Translational Therapeutic Strategies, Hannover Medical School, Hanover, Germany; ^2^Department of Cardiology and Angiology, Hannover Medical School, Hanover, Germany; ^3^Chair of Medical Informatics, Friedrich-Alexander University of Erlangen-Nürnberg, Erlangen, Germany; ^4^Functional Genomics and Systems Biology Group, Department of Bioinformatics, University of Würzburg, Würzburg, Germany; ^5^Institute of Toxicology and Core Unit Proteomics, Hannover Medical School, Hanover, Germany; ^6^REBIRTH Center of Translational Regenerative Medicine, Hannover Medical School, Hanover, Germany; ^7^Department of Internal Medicine III, Cardiology, Angiology and Intensive Care Medicine Martin-Luther-University Halle (Saale), Halle (Saale), Germany

**Keywords:** inflammation, cardiovascular, aging, senescence, smooth muscle cell

## Abstract

The senescence of vascular smooth muscle cells (VSMCs) has been implicated as a causal pro-inflammatory mechanism for cardiovascular disease development and progression of atherosclerosis, the instigator of ischemic heart disease. Contemporary limitations related to studying this cellular population and senescence-related therapeutics are caused by a lack of specific markers enabling their detection. Therefore, we aimed to profile a phenotypical and molecular signature of senescent VSMCs to allow reliable identification. To achieve this goal, we have compared non-senescent and senescent VSMCs from two *in vitro* models of senescence, replicative senescence (RS) and DNA-damage induced senescence (DS), by analyzing the expressions of established senescence markers: cell cycle inhibitors- p16 INK4a, p14 ARF, p21 and p53; pro-inflammatory factors-Interleukin 1β (IL-1β), IL-6 and high mobility group box-1 (HMGB-1); contractile proteins-smooth muscle heavy chain- (MYH11), smoothelin and transgelin (TAGLN), as well as structural features (nuclear morphology and LMNB1 (Lamin B1) expression). The different senescence-inducing modalities resulted in a lack of the proliferative activity. Nucleomegaly was seen in senescent VSMC as compared to freshly isolated VSMC Phenotypically, senescent VSMC appeared with a significantly larger cell size and polygonal, non-spindle-shaped cell morphology. In line with the supposed switch to a pro-inflammatory phenotype known as the senescence associated secretory phenotype (SASP), we found that both RS and DS upregulated IL-1β and released HMGB-1 from the nucleus, while RS also showed IL-6 upregulation. In regard to cell cycle-regulating molecules, we detected modestly increased p16 levels in both RS and DS, but largely inconsistent p21, p14ARF, and p53 expressions in senescent VSMCs. Since these classical markers of senescence showed insufficient deregulation to warrant senescent VSMC detection, we have conducted a non-biased proteomics and *in silico* analysis of RS VSMC demonstrating altered RNA biology as the central molecular feature of senescence in this cell type. Therefore, key proteins involved with RNA functionality, HMGB-1 release, LMNB-1 downregulation, in junction with nuclear enlargement, can be used as markers of VSMC senescence, enabling the detection of these pathogenic pro-inflammatory cells in future therapeutic studies in ischemic heart disease and atherosclerosis.

## Introduction

Inflammation is a hallmark and potent promoter of cardiovascular disease, in addition to other well-defined risk factors that contribute to the multifactorial processes of disease progression (hypercholesterolemia, diabetes, hypertension, and smoking) (Dzau et al., 2002). Elevated inflammatory marker levels, such as interleukin-1β (IL-1β), IL-6, and HMGB-1 are associated with an cardiovascular risk (Ridker, 2016). The CANTOS clinical trial demonstrated that an anti-inflammatory approach through IL-1β blockade improves CV (cardiovascular) outcomes (Ridker et al., 2017). While the results of the trial provide proof of concept that the anti-inflammatory approach provides significant CV benefit, a long-term systemic immunosuppressive therapy highlighted serious concerns about infections and sepsis (Hidalgo and Tall, 2019). Targeting the source and cause of inflammation, rather than individual cytokines may be a solution for these issues.

Cellular senescence has been proposed the key source of inflammation in atherosclerosis and cardiovascular disease (CVD) (Stojanović et al., 2020). Senescent cells are irreversibly cell cycle-arrested and characterized with a pro-inflammatory phenotype known as the senescence associated secretory phenotype (SASP) (Bennett and Clarke, 2016). Experimental evidence showed that the SASP includes several pro-inflammatory cytokines (IL-1β, IL-6, HMGB-1), many of which are known cardiovascular risk factors (Ridker, 2014).

Senescence of various cell types has been implicated in CVD development (Stojanović et al., 2020). In particular, senescent vascular smooth muscle cells (VSMCs) were reported to be one of the key pro-inflammatory senescent cell populations, and were found in unstable, rupture-prone atherosclerotic plaques (Bennett et al., 2016; Uryga and Bennett, 2016; Hamczyk et al., 2018; Katsuumi et al., 2018).

Therapeutic options to target these senescent cells have recently emerged (Stojanović et al., 2020). However, the further development of these strategies and their adaptation for the cardiovascular system is limited by a lack of specific markers that would enable the detection of senescent cell populations. Recent research pointed out toward the limitations of classical senescence markers. Cell cycle inhibitors p16 and p21 were not found to be consistently differentially regulated in senescence of various cell types (Hernandez-Segura et al., 2017). Inconsistencies between patterns of gene and protein expression of these markers were also observed (Marthandan et al., 2016). Additionally, p16 and the senescence-associated β-galactosidase staining (SAβG) levels can be reversibly increased in macrophages (Hall et al., 2017). Most SASP factors can be secreted by immune cells (Stojanović et al., 2020). Moreover, specific markers to detect and differentiate senescent VSMCs from other cell populations are lacking.

To test if the deregulation of previously reported senescence markers applies to senescent human coronary VSMCs, we have performed a detailed characterization of the molecular and phenotypical markers that are common in two separate senescence models *in vitro*, replicative senescence (RS) and DNA-damage-induced senescence (DS). We analyzed classical senescence markers: morphology and structural features of senescent VSMCs cells, cell cycle inhibitor and SASP marker expression, as well as contractile protein expression. To find a more specific signature of VSMC senescence, we conducted an unbiased high-throughput proteomics analysis to reveal novel markers of senescence in VSMCs.

## Materials and Methods

### Chemicals and Antibodies

Bleomycin sulfate was purchased from Enzo Life Sciences (BML-AP302). The following antibodies were purchased: α-Tubulin (Cell Signaling, #2144), ACTA2 (Sigma Aldrich, C6198), β-Actin (Cell Signaling, #4967), HMGB1 (Abcam, ab18256), MYH11 (Abcam, ab53219), Phalloidin-TRITC (Sigma Aldrich, P1951), p16 (Proteintech, 10883-1-AP), p21 (Cell Signaling, #2947), p53 (Santa Cruz, sc6243), smoothelin (Santa Cruz, sc 28562), TAGLN (Abcam, ab14106).

### Cell Culture

Human coronary smooth muscle cells from a young healthy donor were purchased from Promocell (C-12511) and cultured in growth medium (Promocell, C-22062), supplemented with 10% FBS and 1% penicillin-streptomicin (Sigma Aldrich). To achieve RS, cells were serially passaged (Bielak-Zmijewska et al., 2014). Cells were cultured until 70–80% confluence and re-seeded in T75 Flasks (Thermo Fisher Scientific, Nunc EasYFlask, #156499) at a density of 3.5 × 10^3^ cells/cm^2^ until proliferative arrest (passage 6–8). Cumulative population doublings were counted using the formula cPD = X + 3.322^∗^(log Y−log I), X representing the cumulative population doubling of the subculture used to initiate the culture, Y being the viable cell number on harvest, and I the cell number on inoculation. Low passage VSMCs (passage 2–3) were treated with bleomycin to induce DS, as previously reported (Gardner et al., 2015).

### RNA Isolation and RT-qPCR

Gene expressions of p16, p14, p21, IL-1β, IL-6, TNF-α, LMNB-1, and TNFRSF10C were measured through RT-qPCR, and normalized to the mean expression of two housekeeping genes, FBXO7 and GAPDH, as previously suggested (Hernandez-Segura et al., 2019). Total RNA was extracted using the RNeasy Mini Kit (Qiagen, #74104). Reverse transcription was performed using the iScript^TM^ cDNA Synthesis Kit (Biorad, #1708891) and quantitative expression using the iQ^TM^ SYBR^®^ Green Supermix (Biorad, #1708880), utilizing target specific human primers: p16INK4a (Forward 5′-GGGGGCACCAGAGGCAGT-3′; Reverse 5′-GGTTGTGGCG GGGGCAGTT-3′), p14ARF (5′-CCCTCGTGCTGATGCTAC TG-3′; Reverse 5′-CATCATGACCTGGTCTTCTAGGAA-3′), p21 (Forward 5′-TGTCCGTCAGAACCCATGC-3′; Reverse 5′-AAAGTCGAAGTTCCATCGCTC-3′), IL-1β (Forward 5′-GC TGCTCTGGGATTCTCTTC-3′; Reverse 5′-TGCCACTGTA ATAAGCCATCA-3′), IL-6 (Forward 5′-GGCACTGGCAGAA AACAACC -3′; Reverse 5′-GCAAGTCTCCTCATTGAATCC-3′), TNF-α (Forward 5′-GGCGTGGAGCTGAGAGATA-3′, Reverse 5′-CAGCCTTGGCCCTTGAAGA-3′), LMNB-1 (For- ward 5′-AAGCAGCTGGAGTGGTTGTT-3′; Reverse 5′-TTG GATGCTCTTGGGGTTC-3′); TNFRSF10C (Forward 5′-CACC AACGCTTCCAACAATGAACC-3′; Reverse 5′- TCCGGAAG GTGCCTTCTTTACACT-3′), GAPDH (Forward 5′- TGCACC ACCAACTGCTTAGC-3′; Reverse 5′-GGCATGGACTGTGG TCATGAG-3′), FBXO7 (Forward 5′-GCTCGCACCTGAGGC AGTCC-3′; Reverse 5′-GTCTCTTCATCTCCAGTGAG GGG-3′).

### Protein Isolation and Western Blot

Total protein was obtained from cell pellets, which were previously washed with PBS, via the lysis buffer (Biorad). Samples were loaded on 15% polyacrylamide gels for electrophoresis and transferred to a nitrocellulose membrane (Biorad). After blocking for 1 h in 5% milk-tris buffered saline Tween (TBST), membranes were incubated in 5% milk-TBST solution containing the primary antibody overnight. After washing in TBST, the membranes were incubated with the Secondary IgG-HRP secondary antibody (Santa Cruz) for 2 h, ultimately being rinsed and developed by using the enhanced chemiluminescence (ECL) reagent (Biorad).

### Immunofluorescence and Nuclear/Cell Size Measurement

Cells were fixed using the Cytofix Fixation Buffer (BD Biosciences, #554655) for 15 min, washed with Phosphate Buffered Saline (PBS), incubated with blocking buffer for 30 min (Thermo Fisher Scientific, #37515). After adding primary antibodies in blocking buffer, cells were incubated overnight in the dark, washed and stained with secondary anti-bodies Alexa Flour 488 and 546 (Thermo Fisher Scientific). Cells were then washed with PBS and stained with DAPI (Thermo Fisher Scientific). Images were acquired using the Nikon Ni-E fluorescence microscope, with four fields of view per condition, containing at least 300 cells. Nuclear or cell size measurements and nuclear morphometric analysis were performed in Image J software (version 1.6), as described previously (Filippi-Chiela et al., 2012).

### Senescence-Associated β-Galactosidase Staining (SAβG)

The protocol was performed as described by Dimri et al. (1995). Briefly, cells were fixed, washed and incubated in freshly prepared SAβG staining solution (1 mg/ml 5-bromo-4-chloro-3-indolyl-beta-d-galactopyranoside (X-gal), 1 × citric acid/sodium phosphate buffer (pH 6.0), 5 mM potassium ferricyanide, 5 mM potassium ferrocyanide, 150 mM NaCl, and 2 mM MgCl_2_) at 37°C for 16 h. The enzymatic reaction was stopped by washing with cold PBS and cells were counted via bright-field microscopy.

### Liquid Chromatography–Masspectrometry (LC-MS) and Bioinformatical Analysis

LC-MS was performed as described previously (Sonnenschein et al., 2019). Briefly, the whole cell lysate total protein from replicative senescence VSMCs was used for immunoprecipitation and stained via the Coomassie solution for 45min at room temperature. The gel was then de-stained, lanes digested using trypsin and extracted peptides were separated with a LC-MS system (RSLC, Thermo Fisher Scientific, Germany). Raw data was analyzed with MaxQuant software (version 1.5.3.30) and peptides were searched against all human entries of the UniProtKB/Swiss-Prot database via the Andromeda search engine. A false discovery rate of 0.01 on peptide and protein level was used for identification. Data were analyzed using Perseus (version 1.5.2.6) and if applicable two sided one-sample Student’s *t*-test was applied for comparison and visualized via Graph Pad Prism 8. *In silico* analysis of differentially expressed proteins (mean difference >1/<−1) were analyzed using g:Profiler web tool (Raudvere et al., 2019). Functional profiling results were plotted in R (version 3.6.3) using ggplot2 package (version 3.3.0) (Wickham, 2016).

### Statistical Analysis

Results are represented as means ± SEM and were analyzed in Graph Pad Prism 6/8 software. The analysis was performed with the two-sided Student’s *t*-test for unpaired samples and two-way ANOVA for multiple group comparisons. Statistical significance was set at *p* < 0.05.

## Results

### Senescent Coronary VSMCs Have an Enlarged Morphology, but Retain Smooth Muscle Cell Features

**FIGURE 1:**
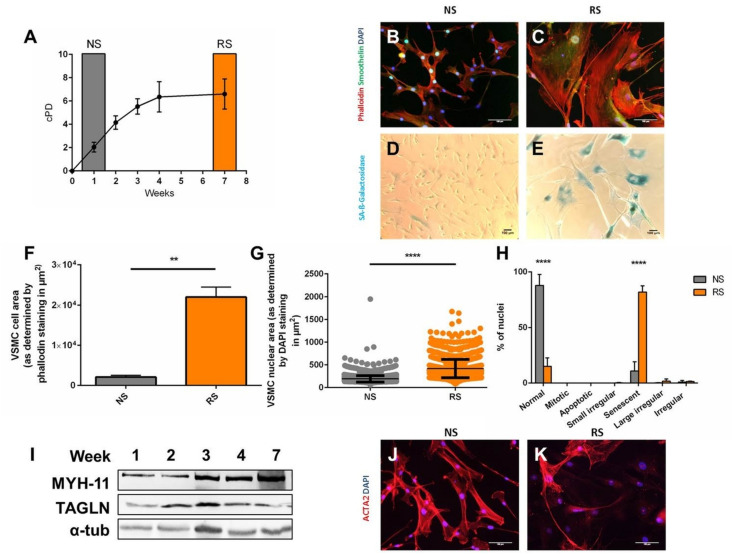
Induction and morphological markers of replicative senescence. Cells were stained DAPI, phalloidin, smoothelin, and α-smooth muscle actin (ACTA2) enabling measurement of cell nuclear size in Image J and characterization. Western blot was per **(A)** Cumulative population doublings (cPD) of serially passaged VSMCs (*n* = 3). **(B,C)** Immunofluorescence microscopy of RS VSMCs, on 20× magnification. **(D,E)** Bright field microscopy of SA-β-galactosidase staining, on 10× magnification. **(F)** Cell size comparison between NS and RS VSMCs, *n* = 3, *t*-test. **(G)** Nuclear size comparison between NS and RS VSMCs, *n* = 3, *t*-test. **(H)** Nuclear morphology categorization via NMA; two-way ANOVA with multiple testing corrections, *n* = 3. **(I)** Western blots of Myosin-11 (MYH-11), Transgelin (TAGLN) and α-tubulin (α-tub) of a passaging timeline in RS. **(J,K)** Immunofluorescence microscopy of ACTA2 in NS and RS VSMCs, on 20× magnification. **p < 0.01, ****p < 0.0001.

To determine if altered morphology can be used to distinguish senescent VSMCs, we induced replicative senescence through serial passaging (Figure 1A) and analyzed non-senescent low passage (NS, week 1 of culture) and replicatively senescent coronary VSMCs (RS, week 7 of culture). NS VSMCs (Figure 1B) showed a spindle-like morphology, typical of contractile VSMCs (Bennett et al., 2016). RS VSMCs (Figure 1C) lost these characteristics, having a flat, enlarged, polygonal morphology. Compared to NS cells (Figure 1D), the increase of size RS cells coincided with SA-β-galactosidase staining positivity (SAβG) (Figure 1E). The quantification of these parameters showed that replicatively senescent cells have an increased cell size (Figure 1F) and enlarged nuclei compared to non-senescent VSMCs (Figure 1G). To confirm that the nuclear enlargement is a consequence of senescence, further analysis conducted via Nuclear Morphometric Analysis (Filippi-Chiela et al., 2012). This high-throughput analysis tool compares size and irregularity of nuclei, enabling the distinction between the morphology of NS nuclei (“normal”) and various other nuclear shapes seen in mitosis, apoptosis and mitotic catastrophe. This analysis confirmed that RS VSMC nuclei are dominantly large and regular, which is a feature of senescent nuclei, excluding other causes of nuclear enlargement (Figure 1H). These findings imply that nuclear morphology can be used as a reliable detection method of senescence, and was therefore used in subsequent experiments. To assess if this abnormal morphology is a consequence of de-differentiation, we observed that some common VSMC markers are retained, as seen on immunofluorescence (smoothelin) and Western blot (MYH11, TAGLN) (Figure 1I). However, RS VSMCs display variable expression of alpha smooth muscle actin (ACTA2) on immunofluorescence (Figures 1J,K).

### Senescent Coronary VSMCs Modestly Upregulate Cell Cycle Inhibitor p16 and Pro-Inflammatory Markers, but Not p14, p21, and p53

**FIGURE 2:**
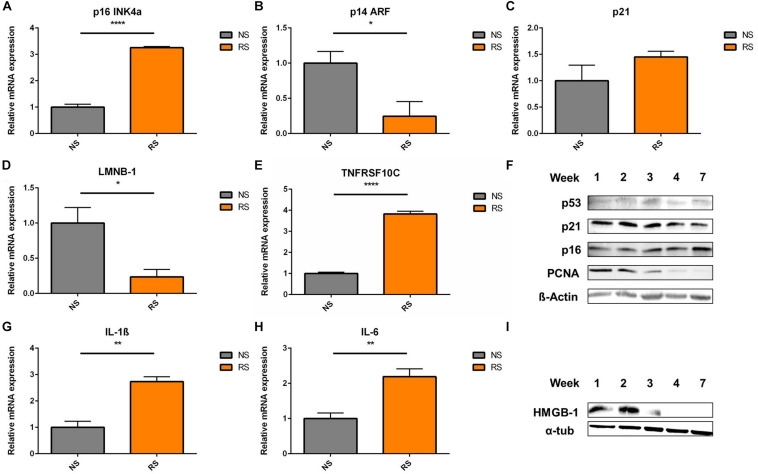
Cell cycle inhibitors and SASP markers in replicative senescence; Expression levels of cell cycle inhibitors **(A)** p16, **(B)** p14, **(C)** p21; **(D)** nuclear envelope protein LMNB-1- Lamin B1 (*n* = 4) and **(E)** TRAIL receptor 3 and tumor necrosis factor receptor superfamily member 10C (TNFRSF10C) interleukins **(G)** IL-1β, **(H)** IL-6- interleukins (*n* = 3), were measured via RT-qPCR, *t*-test; **(F)** shows the Western blots a timeline of p53, p21, p16, and PCNA- Proliferating cell nuclear antigen; α-tub- α-tubulin; **(I)** HMGB-1 (High mobility group box 1) levels in whole cell lysates were analyzed in a passaging timeline until replicative arrest; *p < 0.05, **p < 0.01, ****p < 0.0001.

In terms of cell cycle inhibitors, upregulation of p16 mRNA level was seen in RS VSMCs (Figure 2A). However, p14 was downregulated (Figure 2B) and p21 showed similar expression levels (Figure 2C), when comparing NS to RS, while LMNB-1 was downregulated (Figure 2D) and TNFRSF10C (also known as Decoy receptor 1) was upregulated (Figure 2E), as two additional senescence markers. Analysis at the protein level was conducted on different subsequent passages until cessation of proliferation, as shown by the absence of proliferative cell antigen (PCNA) expression (Figure 2F). p16 showed a mild upregulation trend in the passaging timeline, while p21 and p53 seemed not to be differentially expressed on a protein level (Figure 2F). Senescent VSMCs display a pro-inflammatory phenotype and SASP markers. IL-1β and IL-6 gene expression was increased (Figures 2G,H). HMGB-1 was released from whole cell lysates in the final passages (Figure 2I). When released from the cell, HMGB-1 functions as an aggravating factor in inflammation (Davalos et al., 2013). In conclusion, cell cycle inhibitors were not consistently deregulated and senescent VSMCs possess a pro-inflammatory phenotype. To gain insight into their time-dynamic regulation in another model of senescence, we proceeded to measure their expression levels in a timeline of DNA-damage-induced senescence.

### Senescence Markers Show a Dynamic Development

**FIGURE 3:**
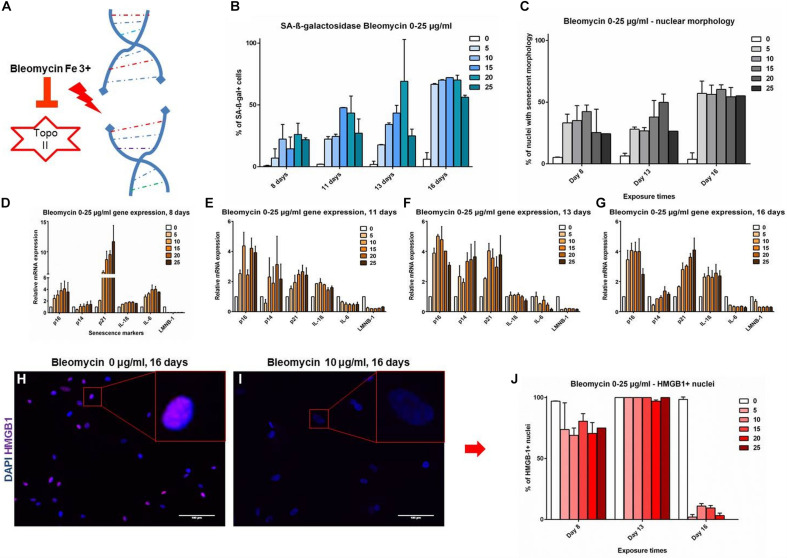
**(A–G)** Senescence markers in DNA damage-induced senescence. Coronary VSMCs were treated with 0–25 μg/ml with bleomycin for 3 h and incubated for indicated time-points (*n* = 2). **(H–J)** Immuno-fluorescence of HMGB-1 localization (*n* = 2). Topo II- Topoisomerase 2; 0-25- bleomycin doses in μg per ml; DAPI- 4’,6-diamidino-2-phenylindole; p16, p14, p21- cell cycle inhibitors; IL-1β, IL-6- interleukins; LMNB-1- Lamin B1; HMGB-1- High mobility group box1.

The markers were further evaluated in a setting of DNA-damage induced senescence (DS) by bleomycin, which induces double strand breaks (Figure 3A; Gardner et al., 2015). An analysis of time and dose-dependent responses in DS revealed a dynamic development of senescence markers. SAβG staining positivity and NMA showed comparable and gradual upward trends, starting at 7.5–25.5% at day 8 and reaching up to 72.9% at day 16, the effect being mostly dose dependant at 0–25 μg/ml bleomycin dose (Figures 3B,C). Cell cycle inhibitors and SASP markers showed a gradual dose- and time-dependant development. It appears that the time-dependence of senescence marker development shows a similar trend across all time points (Figures 3D–G): p16 levels peaked at day 11 and remain stable; p14 is upregulated at intermediate time points (days 11 and 13), but returned to baseline levels on day 16. p21 was strongly upregulated early on day 8, but showed lesser upregulation at day 16; IL-1β was upregulated on day 16, coinciding with HMGB-1 release (Figures 3H–J). IL-6 showed upregulation on day 8, but that evolved into downregulation by day 16; LMNB-1 showed low levels throughout the timeline, with a slight trend of recovery on day 16. The data demonstrates that some senescence markers (p21, p14 and IL-6) can be transiently expressed at different stages of VSMC senescence evolution, without upregulation in late stages. Other markers are more consistently expressed and overlapping with RS (IL-1β, HMGB-1, LMNB-1, nuclear morphology). To find markers that are consistent features of senescent VSMCs in an unbiased, -omics approach, we have analyzed RS VSMCs protein expression with mass spectrometry.

### LC-MS Reveals RNA Metabolism Disturbance as a Key Novel Marker of VSMC Senescence

**FIGURE 4:**
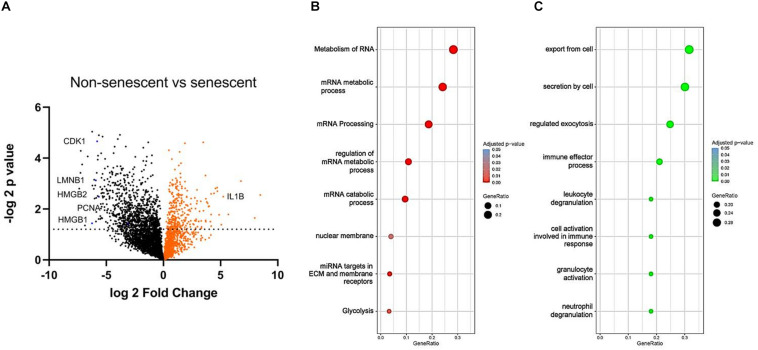
**(A)** Volcano plot of NS and RS VSMC protein expression measured by LC-MS. False discovery rate was set at 0.01, *n* = 3, *t*-test. Gene ontology analysis via the g:Profiler tool of **(B)** downregulated and **(C)** upregulated processes in RS, *t*-test.

To find reliable markers of senescence, we have compared NS (1 week of passaging) to RS (7 weeks of passaging) VSMC protein content with unbiased LC-MS technology (Figure 4A). The expression levels of known markers p16INK4a and p21 were not significantly upregulated, but cyclin dependant kinase 1 (CDK1) and PCNA was low in senescent VSMCs. In terms of other classical markers that were deregulated, IL-1β was upregulated, while LMNB-1 was downregulated. Pro-inflammatory factor HMGB-1, but also of HMGB-2, intracellular protein levels were decreased. We have therefore set out to find potential novel marker candidates among the 1083 significantly differentially regulated proteins in the dataset.

To study the functional changes in senescent cells, we have performed a gene enrichment analysis of the top downregulated (Figure 4B) and upregulated proteins (Figure 4C). Several processes known to be linked to senescence were found, including nuclear membrane abnormalities and inflammation. RNA-related metabolism appeared amongst the most strongly implicated biological processes to be deregulated in senescence, involving a large group of proteins associated with this process (Figure 4B). In addition, RS VSMCs have low glycolytic activity but a high enrichment of secretory processes.

## Discussion

Cellular senescence is defined as an irreversible cell cycle arrest, and is associated with the pro-inflammatory SASP (Stojanović et al., 2020). Senescence has been implicated in multiple chronic diseases, including atherosclerosis, heart failure and aneurism development (Stojanović et al., 2020). To counter these deleterious effects, multiple experimental and therapeutic strategies are currently under development. Due to the limitation of options to detect cellular senescence *in vivo*, these studies utilize mice with reporter genes inserted into the *CDKN2A* gene, which encodes the p16INK4a cell cycle inhibitor (Childs et al., 2016; Schafer et al., 2017). *In vitro* evaluation of senescence in therapeutic studies is often conducted by counting cells positive for SAβG, p21, p53, nuclear enlargement and negative for nuclear HMGB-1 (Jeon et al., 2017; Schafer et al., 2017; Bussian et al., 2018). Recent efforts to improve senescent cell detection revealed that these markers may not be the best option to detect all senescent cell types. p16 and p21 did not universally belong to the senescence signature of various cell lines (Hernandez-Segura et al., 2017). A single cell qPCR study showed that p16 is not the most reliable features of fibroblast senescence on a cell to cell basis (Wiley et al., 2017). Possible explanations for these issues include cell type specificity of the senescence machinery (Hernandez-Segura et al., 2017), dependency on the causative stimulus (Olivieri et al., 2018), the transient nature of the expression of some markers (Hernandez-Segura et al., 2017), and differences between expression levels on the mRNA and protein level (Marthandan et al., 2016).

In our study, we aimed to characterize the senescence of vascular smooth muscle cells, as these cells have been implicated in promoting atherosclerotic disease through the secretion of pro-inflammatory factors (IL-1β, IL-6, HMGB-1) and are thus an interesting anti-inflammatory therapeutic target (Stojanović et al., 2020).

We could show that senescent VSMCs demonstrate nuclear enlargement and cell morphology abnormalities in both RS and DS, a feature of senescence found in various cell types (Filippi-Chiela et al., 2012; Ogrodnik et al., 2017). In line with the nuclear abnormalities, senescent VSMCs in both RS and DS show low LMNB1 levels, from the earlier time points in DS. However, nucleomegaly takes more time to fully develop and seems to occur at intermediate to late phases of DS development *in vitro.* Whether LMNB1 abundancy is actively regulated (transcriptionally or post-transcriptionally) remains unclear.

VSMCs are known to undergo a phenotypic switch from a contractile to a secretory phenotype (Bennett et al., 2016). Highly passaged VSMCs were reported to lose smooth muscle cell contractile markers (Bennett et al., 2016). We indeed observe decreased and disorganized ACTA2 expression in RS on immunofluorescence, as well as active secretory processes *in silico*. However, some RS cells remain positive for ACTA2. Furthermore, RS VSMCs retain MYH11 and TAGLN expression, which is also consistent with previous studies for TAGLN (Miao et al., 2017). These results are in line with recent research showing heterogeneity of senescent cells (van Deursen, 2014; Olivieri et al., 2018; Tang et al., 2019), and warrant future study on a single-cell level (Olivieri et al., 2018).

**FIGURE 5:**
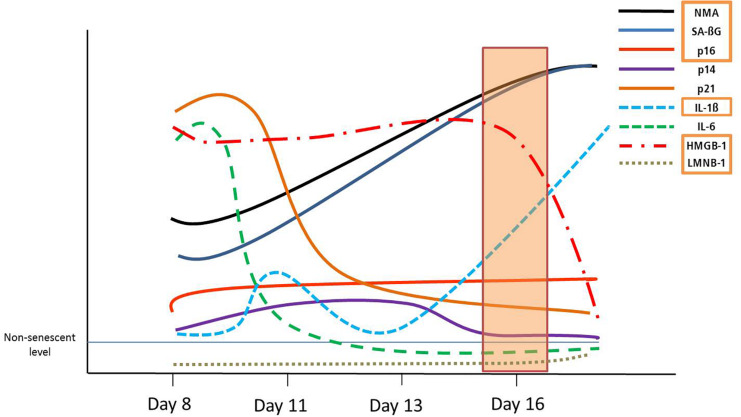
Graphical summary of the dynamic development of senescence markers in DS. Orange rectangles signify markers shared with RS, from day 16.

Senescent VSMCs show a complex cell cycle footprint, with low PCNA and CDK1 levels, that indicate a lack of proliferation. Cell cycle inhibitor expression includes consistently increased p16 levels on a mRNA, and a time-dependant dynamic p14/p21/p53 regulation. These dynamic trends in DS are schematically represented in Figure 5. In addition, p14, p21 and p53 are key regulators of quiescence (Hernandez-Segura et al., 2017), which may make the detection of senescent cells less specific.

Our data at an mRNA (RT-qPCR) and protein level (LC-MS) shows that senescent VSMCs upregulate several pro-inflammatory factors. IL-1β is overexpressed in both RS and DS, while IL-6 seems to be upregulated in RS and “early-phase” DS. HMGB-1and HMGB-2 are key regulators of the SASP, and show downregulated intracellular levels in senescence (Davalos et al., 2013; Guerrero and Gil, 2016). HMGB-1 nuclear release to the extracellular space has been implicated as another mechanism of the inflammation activation by senescent cells, where it acts as an alarmin that promotes the response of innate immunity (Davalos et al., 2013; Guerrero and Gil, 2016). In both RS and DS, VSMCs released this factor consistently. Indeed, *in silico* functional analysis confirmed that senescent VSMCs show a pro-inflammatory phenotype that promotes innate immunity activation. Therefore, we could confirm that senescent VSMCs are a source of inflammatory factors. Hypothetically, senescent VSMCs may be able to persistently self-activate inflammation in an autocrine and paracrine manner through an IL-1α – IL-1β – HMGB1 feedback loop. Others have reported that the IL-1α protein acts as a key driver of the SASP in senescent VSMCs (Orjalo et al., 2009; Gardner et al., 2015). IL-1α can aid IL-1β production by promoting IL-1-receptor signaling (Di Paolo and Shayakhmetov, 2016). HMGB-1 also triggers IL-1β release in VSMCs (Kim et al., 2018). Although we could not find IL-1α deregulation in our dataset, it still may be a key intermediary in this process, independently of its expression levels. Such mechanisms may explain the multimodal contribution of senescent VSMCs to chronic systemic and vascular inflammation, and are thus a valid therapeutic entry point (Stojanović et al., 2020).

Based on results above, it appeared that not all classical senescence markers display sufficient consistency and differential regulation to serve as reliable detection tools for senescent VSMCs. To tackle this issue, we have generated a proteomics dataset from RS samples. We could find global RNA metabolism processes highly enriched in our proteomic dataset, with mRNA splicing, transport and catabolism being significantly affected. Indeed, altered RNA splicing has been reported in senescence for specific molecules, such as p53 (Deschenes and Chabot, 2017). This mechanism may explain the discrepancy between expression levels of p16 on an mRNA and protein level, as RNA-binding proteins are known to regulate tumor suppressor expression at a pre-translational level (Wang et al., 2005; Majumder et al., 2016). Additionally, the amino-acid sequence based detection in LC-MS does not allow the detection of post-translational modifications of proteins (Frescas et al., 2017), which may be a limiting factor in detection. These observations need to be studied further, but may be an important point of considerations in studies encountering issues with p16INK4a detection (Marthandan et al., 2016; Hernandez-Segura et al., 2017; Wiley et al., 2017). Nevertheless, altered proteins involved in RNA metabolism appear to be a key feature of VSMC senescence and may be used as markers to detect these cells.

In summary, we found that VSMCs display an atypical molecular signature that only partially adheres to the commonly used senescence markers. Altered RNA metabolism is a consistent feature of senescent VSMCs, potentially facilitating their detection in future mechanistic and therapeutic studies.

## Data Availability Statement

All datasets generated for this study are included in the article/Supplementary Material, further inquiries can be directed to the corresponding authors.

## Author Contributions

SS, MF, MK, KX, AJ, AP, JB, JF, DS, and TT contributed to the conception and design of the study. SS performed expression and microscopy experiments and wrote the first draft of the manuscript. AJ and AP performed the mass spectrometry. SS, MF, MK, KX organized the proteomics database. SS, MF, MK, KX, AP, and JF performed the statistical and *in silico* analysis. SS and MF wrote sections of the manuscript. DS and TT equally contributed in the supervision of the process. All authors contributed to manuscript revision, read and approved the submitted version.

## Conflict of Interest

TT has filed and licensed patents regarding non-coding RNAs in CVD. TT was the founder and shareholder of Cardior Pharmaceuticals GmbH. The remaining authors declare that the research was conducted in the absence of any commercial or financial relationships that could be construed as a potential conflict of interest.

## Supplementary Material

TABLE S1 Proteomics dataset of NS vs RS VSMCs.
